# Delivering Personalized Recommendations to Support Caregivers of People Living With Dementia: Mixed Methods Study

**DOI:** 10.2196/35847

**Published:** 2022-05-03

**Authors:** Jinhee Cha, Colleen M Peterson, Ashley N Millenbah, Katie Louwagie, Zachary G Baker, Ayush Shah, Christine J Jensen, Joseph E Gaugler

**Affiliations:** 1 Medical School and School of Public Health University of Minnesota Minneapolis, MN United States; 2 University of Michigan Transportation Research Institute University of Michigan Ann Arbor, MI United States; 3 Division of Health Policy and Management School of Public Health University of Minnesota Minneapolis, MN United States; 4 Riverside Center for Excellence in Aging and Lifelong Health Williamsburg, VA United States

**Keywords:** caregivers, caregiving, Alzheimer, dementia, intervention, COVID-19

## Abstract

**Background:**

Estimates suggest that 6.2 million Americans aged ≥65 years are living with Alzheimer dementia in 2021, and by 2060, this number could more than double to 13.8 million. As a result, public health officials anticipate a greater need for caregivers of persons with Alzheimer disease or related dementia and support resources for both people living with dementia and their caregivers. Despite the growing need for dementia caregiver support services, there is a lack of consensus regarding how to tailor these services to best meet the heterogeneous needs of individual caregivers. To fill this gap, *Care to Plan* (CtP), a web-based tool for caregivers of people living with dementia, was developed to provide tailored support recommendations to dementia caregivers.

**Objective:**

The aim of this study is to formally explore the feasibility, acceptability, and utility of CtP for 20 family members of people living with dementia within a health system over a 1-month time period using a mixed methods parallel convergent design.

**Methods:**

A moderately sized health system in the mid-Atlantic region was selected as the site for CtP implementation, where 20 caregivers who were family members of people living with dementia were enrolled. The web-based CtP tool was used by caregivers and facilitated by a health care professional (ie, a *senior care navigator* [SCN]). Caregivers were given a 21-item review checklist to assess barriers and facilitators associated with reviewing CtP with an SCN. Following the 21-item review checklist, semistructured telephone interviews, which included 18 open-ended questions, focused on the facilitators of and barriers to CtP implementation and recommendations for future implementation.

**Results:**

Quantitative results suggested that 85% (17/20) of caregivers indicated that CtP was helpful and 90% (18/20) would recommend CtP to someone in a similar situation. The qualitative analysis identified 4 themes regarding facilitators of and barriers to implementation: caregiver factors, SCN factors, CtP tool system factors, and recommendations and resources factors.

**Conclusions:**

CtP was found to be not only feasible but also a valuable tool for caregivers seeking resources for themselves and their people living with dementia. Long-term evaluation findings aim to generate results on how CtP can be integrated into care plans for caregivers and how SCNs can provide additional support for caregivers of people living with dementia over an extended period.

## Introduction

### Background

In 2019, more than 16 million family members and other unpaid caregivers provided care for people living with dementia [1]. Current estimates suggest that 6.2 million Americans aged ≥65 years are living with Alzheimer dementia in 2021, and by 2060, this number could more than double to 13.8 million [1]. Consequently, public health officials anticipate a greater need for caregivers of persons with Alzheimer disease or related dementia (ADRD) and resources to support these caregivers and their care recipients. Caregivers of people living with dementia often experience physical, emotional, physiological, and financial challenges [2-4]. Caregivers who experience excessive burden are at an increased risk of mood disorders, cognitive decline, cardiovascular diseases, and other ailments that decrease their health [3]. Compounding their burden, caregivers may lack quality information about support strategies that can alleviate the potential challenges of dementia care [5].

Among interventions designed to assist caregivers, strategies where caregivers are actively involved with the intervention and that feature tailored and flexible support systems appear most effective [6]. A systematic review by Hodgson and Gitlin [7] identified more than 200 randomized controlled trials to support caregivers of people living with dementia. Among these interventions, the use of innovative technologies to educate and support caregivers is an emerging area of interest [8,9]. In addition, individualized care counseling, as opposed to group interventions, has demonstrated better outcomes in managing caregivers’ distress [10]. However, few interventions developed for caregivers of people living with dementia have been implemented in practice [11]. A recent review found that only 6 out of 200 efficacious studies for caregivers of people living with dementia have been translated into practice [6]. Moreover, there is a lack of consensus on how to tailor and deliver these services to caregivers of people living with dementia to meet their diverse needs. For example, dementia care needs often vary because of kin relationship with the care recipient, dementia stage, and perceived stress related to dementia care provision [6].

To fill this gap, *Care to Plan* (CtP), a web-based tool for caregivers of people living with dementia, provides tailored support recommendations along with additional guidance from a care navigator (ie, health professional) who can assist caregivers in completing the web-based tool and discussing the CtP’s individualized recommendations [12]. In developing the prototype of the CtP, 21 caregivers of people living with dementia were recruited to test the feasibility and utility of the CtP tool. Following the prototype testing, stakeholders, including professionals, community advocates, and family caregivers of people living with dementia, were recruited to form a community advisory board. This board reviewed the tool’s language and improved its user-friendliness [5]. The function, usability, and clarity of the CtP prototype were positively appraised in a multiphase pilot testing process that included 30 dementia caregivers [12].

### Objectives

The objective of this study was to formally explore the feasibility, acceptability, and utility of CtP for 20 family members of people with ADRD within a health system over a 1-month period using a mixed methods approach. This approach contributes to the evidence base of CtP by determining the implementation potential of this web-based tool in an actual health care system that offers support and services for caregivers of people living with dementia within their system. The quantitative and qualitative evaluation of CtP will provide insights into subsequent refinement of CtP, a more extensive evaluation of efficacy, and efforts to effectively disseminate and implement CtP within health care systems or similar real-world contexts.

## Methods

### Site Description

A moderately sized health system in the mid-Atlantic region was selected as the site for the CtP implementation. The health system represents more than 600 physicians and advanced practice providers offering services and programs in prevention, primary care, diagnostics, neurosciences, oncology, orthopedics, aging-related services, rehabilitation, medical education, home care, and hospice. The system’s network serves approximately 2 million individuals annually, from primary care clinics to residential long-term care facilities.

The health care system uses a senior care navigator (SCN) program, where 3 SCNs provide phone support for older adults and their family caregivers. All the 3 SCNs are certified dementia practitioners and certified senior advisors. One SCN is a licensed practical nurse who has served as an SCN for more than 20 years. The other 2 SCNs have completed graduate work in gerontology. Moreover, all SCNs have experience in supporting residents in long-term care communities, with a focus on serving those who reside at home. Through the SCN program, SCNs connect with callers to provide a variety of support services, including medication management, transportation, meals, and behavioral health help. The CtP tool was incorporated into the health care system’s SCN program to integrate itself into SCN consultation routines with caregivers. The prior professional relationship established between the principal investigator of the CtP and a center director within this health care system increased the feasibility of rapid and efficient CtP implementation at this site. Before the launch of the CtP, all 3 SCNs were selected to test the use of the CtP tool, participate in project meetings, and contribute to the promotion of CtP.

### Recruitment

A total of 20 caregivers who were family members of people living with dementia were enrolled. Sources of recruitment included an SCN case management program, a geriatric assessment clinic, a memory care clinic, a memory café program, an evidence-based caregiver intervention program, community webinars or educational events, the health care system’s Intranet, approved flyers, advertisements, emails, and social media. Caregivers who were interested and agreed to be contacted about the study were referred to the research team for enrollment.

### Ethics Approval

The study reported in this paper was approved by the University of Minnesota Institutional Review Board (approval number: STUDY00005971).

### Inclusion and Exclusion Criteria

The following inclusion criteria were applied to potential participants: (1) the care recipient had a provider diagnosis of ADRD; (2) the caregiver was aged >21 years; (3) the participant was English-speaking; (4) the participant self-identified as someone who provides help to people living with dementia because of their cognitive impairments; (5) the participant indicated a willingness to use CtP; and (6) the participant resided in 1 of the 4 regions serviced by the health care system (based on zip code). Those who did not meet these criteria were not eligible. In addition, those who endorsed a history of a serious mental health disorder whose (1) symptoms were exacerbated in the last 6 months and (2) were not receiving steady, ongoing pharmacological or other treatment for these symptoms were excluded from the project.

### Design

This study examined the use of CtP, a free web-based care planning tool that generates individualized service recommendations for caregivers of people living with dementia. A convergent parallel mixed methods design (quantitative + qualitative) was implemented to examine the feasibility, acceptability, and utility of CtP over a 1-month period [13]. Baseline and follow-up data were collected via telephone, with the exception of 1 participant who requested a hard copy through mail.

Following enrollment and an initial survey, an SCN contacted the caregiver to guide them through the CtP via telephone. SCNs placed particular emphasis on helping caregivers understand the recommended resources provided. A 21-item multiple-choice assessment (the CtP Review Checklist; Table S1 of Multimedia Appendix 1) was collected at 1 month after enrollment by the UMN research staff. Following the CtP Review Checklist administration, caregivers were asked to complete a semistructured telephone interview about their experience with the CtP tool, which lasted approximately 30 to 45 minutes. The 3 SCNs also participated in semistructured interviews to obtain their perceptions when administering CtP.

### Intervention

A team of SCNs from the health care system collaborated with the UMN research team to identify resources and contacts in the 4 geographic regions served by their health care system. The web-based CtP tool is located on a secure platform and can be used directly by caregivers or facilitated by an SCN [14]. In this study, all caregivers used the CtP tool together with an SCN because feedback from the original CtP development study suggested that caregivers preferred this human guidance when using the web-based tool [15]. CtP was also designed to be user-friendly and featured visual cues and videos for navigating the tool (see Multimedia Appendix 1 for figures of CtP).

CtP includes a 20-item assessment, administered over the phone with an SCN, specifically designed to determine caregivers’ needs and match them with resources that might help. Items on the assessment were based on 6 dimensions of the validated Risk Appraisal Measure linked to caregiver risk and amenable to intervention: depression, burden, self-care and health behaviors, social support, safety, and patient problem behaviors [12]. Risk for caregiver distress and their care recipient’s risk for nursing home admission were also assessed based on several contextual characteristics [15]. Following the completion of the brief assessment, CtP generated an individualized support recommendation based on their responses (for details on how individualized responses were matched to support service recommendations, see the studies by Gaugler et al [15,16]). Caregivers received region-specific resources based on their zip codes and tailored recommendations based on assessment responses in seven categories: (1) skills building (ie, educational programs), (2) problem solving (ie, care consultation), (3) changing your thinking (ie, therapy), (4) taking a break (ie, respite), (5) brain health (ie, exercise and meditation), (6) support groups, and (7) high-powered combinations (ie, evidence-based multicomponent programming; see Multimedia Appendix 1 for figures of CtP). More than 30 resources were incorporated into the tool, with an average of 12 per each of the 4 regions. Example resources include the local Area Agencies on Aging, the Alzheimer’s Association, and other local agencies or programs. Recommendations were developed based on clinical expert recommendations from 422 clinical professionals and scientific experts from across the United States [15]. Information on the resources provided to caregivers over the telephone by the SCNs was also later mailed by the research staff.

### Data Collection

#### Measures: Context of Care

Demographics and context of care variables were collected at baseline for caregivers of people living with dementia and included gender, age, race, ethnicity, marital status, number of living children, income, employment, relationship to care recipient, and education. Caregiver’s residence and Medicaid coverage status were also collected at baseline. To remain consistent with the caregiving literature and other CtP publications, income cutoffs were different between caregivers of people living with dementia and people living with dementia [14,15,17].

#### Measures: Objective Stressors

Primary caregiver objective stressors (eg, dementia severity among care recipients) were also collected for caregivers of people living with dementia. These stressors included dependence of people living with dementia on their caregiver to complete 6 activities of daily living (ADL) [18] and 6 instrumental ADL [19]. An 8-item memory impairment scale assessed the intensity of memory loss, communication deficits, and recognition of impairment at each time point of people living with dementia [20]. The frequency and level of ADRD-related behavioral problems were measured using the Revised Memory and Behavior Problem Checklist [21].

#### Measures: Caregiver Outcomes

Caregiver self-efficacy was measured using 8 items examining participants’ certainty that they could carry out specific behaviors related to dementia care [22]. Caregiver distress was measured using the 20-item Center for Epidemiological Studies-Depression (CES-D) scale [23]. Three additional measures of caregiver distress were included: a 5-item measure of role overload, a 5-item measure of role captivity, and a 5-item measure of loss of intimate exchange [20].

#### CtP Review Checklist

Approximately 1 month after using CtP with an SCN, caregivers were administered a 21-item review checklist to assess barriers and facilitators associated with using CtP with an SCN. The 21-item review checklist was specifically designed to test the feasibility and utility of CtP. Its design and creation have been described previously [15]. Items on the 21-item checklist were administered as a 5-point Likert scale ranging from strongly disagree to strongly agree and a *not applicable* option. An example item in the checklist was “I would recommend CtP to others in a similar situation.” A full list of the items is presented in Table 1 (Cronbach *α*=.90). Following the 21-item review checklist, caregivers participated in 30-minute semistructured telephone interviews. Open-ended questions focused on facilitators and barriers to CtP implementation and use and recommendations for future implementation. Example items include “What were some of the factors that made CtP easy to use?” “What were some of the factors that made CtP difficult to use?” (see Multimedia Appendix 1 for all base questions). The interviews were digitally recorded and transcribed later.

**Table 1:** One-month Care to Plan (CtP) Review Checklist scores (Likert-type scale: 1=strongly disagree, 2=disagree, 3=feel neutral, 4=agree, and 5=strongly agree).

	Value, mean (SD)	Agree and strongly agree, n (%)
It was easy to review the CtP tool with (health system).	4.70 (0.80)	16 (80)
The information provided by the Senior Care Navigator was clear to me.	4.55 (0.76)	17 (85)
The questions I answered during the CtP assessment were easy to understand.	4.35 (0.75)	19 (95)
I was able to understand the services recommendations provided by CtP.	4.50 (0.61)	19 (95)
The person guiding me through CtP was helpful.	4.55 (0.60)	19 (95)
I valued having a Senior Care Navigator available to discuss the recommendations from CtP.	4.35 (0.59)	19 (95)
After using CtP, I was able to find a service that looks as though it will meet my needs.	4.35 (1.35)	13 (65)
After using CtP, I was able to find a service that looks as though it will meet my relative’s needs.	4.95 (1.36)	11 (55)
There are financial constraints to me being able to use the services recommended by CtP^a^.	4.05 (1.67)	8 (40)
There are time constraints to me being able to use the services recommended by CtP^a^.	3.50 (1.79)	9 (45)
I am planning on using a service recommended by CtP.	4.15 (1.42)	13 (65)
The care navigator helped me contact a service recommended by CtP.	4.30 (1.75)	9 (45)
CtP was helpful.	4.35 (0.75)	17 (85)
CtP could be improved^a^.	3.25 (1.48)	6 (30)
I wish I would have completed CtP sooner.	3.95 (1.28)	10 (50)
Transportation issues make it unlikely that I will be able to use the recommendations provided by CtP^a^.	4.65 (1.09)	17 (85)
CtP provided me with a sufficient number of options to support me.	4.05 (1.23)	14 (70)
CtP provided me with a sufficient number of options to support my relative.	4.50 (1.36)	15 (75)
The resources recommended by CtP were new to me.	3.00 (1.21)	9 (45)
I would recommend CtP to others in a similar situation.	4.45 (0.69)	18 (90)
I would use CtP again in the future.	4.15 (0.93)	15 (75)
Summary score	4.22 (0.69)	N/A^b^

^a^Item reverse coded.

^b^N/A: not applicable.

### Data Analysis

#### Quantitative Analysis

Authors JC and CMP conducted a quantitative descriptive analysis of the baseline descriptive characteristics of caregivers and people living with dementia. These authors also completed Kendall Tau-B and Spearman bivariate correlations. Bivariate correlations were conducted for the following measurements to identify subcategories of caregivers that benefited more or less: CtP Review Checklist scores, baseline context of care, primary objective stressors (ADL, instrumental ADL, Memory Impairment, and Revised Memory and Behavior Problems Checklist-Frequency), resources (caregiver self-efficacy) and caregiver distress (CES-D, role overload, and role captivity loss of intimate exchange). In addition, the percentage of agree and strongly agree responses was calculated for individual items from the CtP Review Checklist scores to measure the agreeableness of that item. An additional variable that summed up and averaged all 21 items in the CtP Review Checklist for each participant was created as a summary measure of the feasibility, usability, and perceptions of CtP. All quantitative data were analyzed with SPSS (version 24; IBM Corp) [24].

#### Qualitative Analysis

Authors JC, CMP, ANM, KL, ZGB, and CJJ open coded all qualitative data from the semistructured telephone interview about caregivers’ experience with the CtP tool. We followed thematic analysis best practices as described by Braun and Clarke [15] to code themes collected for qualitative analysis. All coders read a different subset of transcripts at random and generated preliminary coding categories based on common ideas they identified in the data. Thereafter, coders met regularly to discuss their preliminary codes to refine and adjust their codes to best portray the data as a whole. Disagreements in codes were resolved by consensus. Authors JC and CMP reviewed the codes to discern overarching themes, which were then reviewed and agreed upon by all the authors [25]. Regular debriefings discussed the interpretation and overarching themes and led to saturation, as described by Dey and Saunders et al [26,27]. All quantitative data were analyzed using NVivo (version 12) [28].

## Results

### Sample Characteristics

Sample characteristics of caregivers are presented in Table 2. Caregivers were primarily White (15/20, 75%), married (17/20, 85%), and female (18/20, 90%) with a mean age of 66.7 (SD 11.43) years (Table 2). Most caregivers also had less than a bachelor’s degree (13/20, 65%) and a total household income of at least US $80,000 (10/20, 50%; Table 2). Slightly over half of the caregivers in our study who used CtP were the spouse or partner of the people living with dementia (11/20, 55%), followed by adult children (7/20, 35%). Caregivers reported varying levels of caregiver distress (based on their Revised Memory and Behavior Problems Checklist-Reaction, loss of intimate exchange, role captivity, role overload, and CES-D) and resources (self-efficacy). Notably, the average caregiver score exceeded the CES-D cutoff of 16, indicating depression (mean 16.95, SD 10.69) [17].

The characteristics of the people living with dementia are presented in Table 3. People living with dementia were primarily White (15/20, 75%), married (13/20, 65%), and male (12/20, 60%) with a mean age of 80.16 (SD 7.91) years. Most people living with dementia had less than a bachelor’s degree (3/10, 15%, with a bachelor’s degree or higher) and had a total household income of at least than US $30,000 (16/20, 80%).

**Table 2:** Caregiver baseline descriptive characteristics (N=20).

Caregiver demographics		Values	Spearman rho correlation with Care to Plan summary score (*P* value)
Female, n (%)		18 (90)	.50
Age (years), mean (SD)		66.68 (11.43)	.82
White population, n (%)		15 (75)	.90
Married, n (%)		17 (85)	.84
Number of living children, mean (SD)		2.25 (1.55)	.98
Annual income of ≥US $80,000, n (%)		10 (50)	.46
Employed, n (%)		7 (35)	.64
Spouse of people living with dementia, n (%)		11 (55)	.19
Adult child of people living with dementia, n (%)		7 (35)	.46
Bachelor’s degree (4-year college) and higher, n (%)		7 (35)	.10
**Primary objective stressors, m** **ean (SD)**			
	ADL^a^ dependencies	2.40 (2.77)	.50
	IADL^b^ dependencies	9.00 (3.11)	.53
	Memory impairment	21.10 (5.06)	.40
	RMBPC-F^c^	9.85 (4.41)	.88
**Resources, mean (SD)**			
	Caregiver self-efficacy	28.10 (6.29)	.09
**Caregiver distress, mean (SD)**			
	CES-D^d^	16.95 (10.69)	.25
	RMBPC-R^e^	15.85 (12.03)	.46
	Loss of intimate exchange	5.50 (2.21)	.96
	Role captivity	6.70 (3.36)	.16

^a^ADL: activities of daily living.

^b^IADL: instrumental activities of daily living.

^c^RMBPC-F: Revised Memory and Behavior Problems Checklist-Frequency.

^d^CES-D: Center for Epidemiological Studies-Depression.

^e^RMBPC-R: Revised Memory and Behavior Problems Checklist-Reaction.

**Table 3:** Baseline descriptive characteristics of people living with dementia (N=20).

Demographics of people living with dementia	Value
Female, n (%)	8 (40)
Age (years), mean (SD)	80.16 (7.91)
White population, n (%)	15 (75)
Married, n (%)	13 (65)
Number of living children, mean (SD)	2.85 (1.66)
Bachelor’s degree or higher, n (%)	3 (15)
Annual income of ≥US $30,000, n (%)	16 (80)
Lives with a caregiver, n (%)	14 (70)
On Medicaid, n (%)	3 (15)

### Empirical Associations Between 1-Month CtP Checklist and Other Domains

There were no statistically significant (*P*<.05) correlations between the CtP Review Checklist summary score and caregiver context of care, objective stressors, or distress achieved statistical significance.

### Mixed Qualitative and Quantitative Results

Qualitative analyses identified 4 themes regarding facilitators of and barriers to the implementation and use of CtP within four overarching categories: (1) caregiver factors, (2) SCN factors, (3) CtP tool system factors, and (4) recommendations and resources factors. Facilitators in each category are discussed later, followed by the barriers. A summary of all the themes, facilitators or barriers, their descriptions, and supporting quotes can be found in Table S2 in Multimedia Appendix 1. Relevant item-level quantitative results from the CtP Review Checklist complemented our quantitative results to offer a more robust description of our themes (Table 1).

### Facilitators to CtP Implementation and Use: Caregiver Factors

Caregivers found that the tool appropriately tailored recommendations to their needs and context of care. This was consistent with the analysis of the 1-month CtP Review Checklist, in which 65% (13/20) of caregivers agreed or strongly agreed that, after using the tool, they were able to find a service that would meet their needs and 55% (11/20) of their people living with dementia needs (Table 1). Giving names to categories of need helped some caregivers better conceptualize their caregiving for both themselves and their people living with dementia. One caregiver noted, “It made me think of different things that I hadn’t previously thought of” (Wife, aged 71 years). Another caregiver explained:

It broke things down to different types of situations and needs. And in some ways even though I’ve been living with this, it helped me better understand my situation and my husband’s situation...It seemed very thorough, and a logical progression and dealt with not just my husband and his needs, but with me and my needs.

Wife, aged 76 years

The helpfulness of the CtP was a recurring theme in the qualitative results. Caregivers said that they enrolled in the study because they were overwhelmed and needed help. The aforementioned caregiver pointed out, “I didn’t know what I didn’t know” and “(I) can use all the help I can get” (Wife, aged 76 years). The helpfulness of CtP was also reflected in the CtP Review Checklist, in which 85% (17/20) of the caregivers agreed or strongly agreed that CtP was helpful (Table 1). CtP was also a source of help that caregivers of people living with dementia could turn to with the help of an SCN. SCNs spoke with caregivers during a time of potentially overwhelming need, and predefined but nuanced categories could simplify how to route caregivers to the help they needed. For example, CtP could:

Help to better define the challenges that caregivers are faced with, that family members are faced with. I think it helps to really provide the proper channeling of resources in the right categories.

SCN

Overall, 90% (18/20) of the caregivers reported that they would recommend CtP for others in a situation similar to them and 75% (15/20) would use CtP again (Table 1).

### Facilitators to CtP Implementation and Use: SCN Factors

Many caregivers appreciated having an SCN available to guide them through the CtP tool. Qualitative analyses highlighted that caregivers appreciated their SCN’s familiarity with caregiving support and the ability to explain the available resources. For those who were new to caregiving for people living with dementia, connecting with someone who was knowledgeable in these areas was especially useful. One caregiver explained how having:

Someone who can speak to it personally even though it might not have been exactly the situation in our household, just somebody who totally gets it and how life-changing it is, not just for the person but for the whole family...I really appreciated that part.

Daughter, aged 58 years

Even those who did not find an absolute need for the SCN liked knowing there was someone they could go to should the need arise: “I like the idea that there’s someone there. I haven’t really found it necessary to use a navigator, per se. Again, I think it’s because I was so early in the situation” (Wife, aged 71 years).

Caregivers also noted that scheduling CtP with an SCN held them accountable for using the tool, even if it was just via a telephone conversation. Although caregivers could have used the CtP website themselves, some admitted that, with competing demands on their attention, they might not have actually used the tool. One caregiver noted, “I think it was just the time it was going to take to do it, and I think that was my problem” (Wife, aged 74 years). In addition, caregivers acknowledged that the SCN could discuss barriers to enacting CtP recommendations and hold them accountable for their own self-care without judgment. Another study recognized how SCNs helped prioritize self-care while also guiding them through resources:

I actually appreciated the Care Navigator...Just that sometimes when you’re so overwhelmed by everything that’s going on, even though you’re a big person, you still need somebody to kind of take you by the hand and say “Let’s get through this. Let’s walk through this and kind of just help you focus.”

Daughter, aged 56 years

Finally, having SCNs go through the CtP tool added a personal touch for caregivers. Because of their background, SCNs could empathize with caregivers’ situations and make themselves available as emotional pillars of support. One caregiver said that the SCN “could share experiences of her own that would make this helpful. So, it was great talking with her” (Daughter, aged 57 years). Another study explained in detail how SCNs’ experiences made them allies:

It’s that connection of somebody who kind of understands personally what you’re going through, kind of no judgement, someone I felt comfortable enough to share some very candid things with, just the overall frustration and grief and loss you feel. Yeah. So, I thought that was really helpful to have another person who really totally has walked through these steps and several steps ahead. That’s always very helpful.

Daughter, aged 52 years

The rapport and personal connection were amplified by the fact that SCNs often had personal caregiving experiences of their own. They could recommend the same resources that helped them:

I think all the content that’s in there is quite pertinent, and I think it’s information that individuals are really going to need, and I base my answer or comments on the fact that personally I’ve had to go through this experience of caregiving with my mom, dad and my aunt, and I’ve actually used the resources, so now as a professional when I’m suggesting the resources and I’m hearing familiar stories of people that are going through struggles with caregiving I can share with them that they’re at the right place getting this information.

SCN

The CtP Review Checklist also reflected how useful caregivers found their SCNs: 85% (17/20) of the caregivers either agreed or strongly agreed that the information provided by the SCN was clear to them, and 95% (19/20) valued having an SCN available to discuss the recommendations from CtP (Table 1).

### Facilitators to CtP Implementation and Use: CtP System Factors

Among the caregivers who described themselves as *tech savvy*, most caregivers found the website interface user-friendly and navigated CtP with ease. Some caregivers even alerted the staff to glitches while moving through the recommended resource pages, which were fixed early on in the project. Although some found that having an SCN helped set time aside in their busy schedules to go through the tool, others found that being able to use the tool on their own worked better for their hectic schedules. Participants could complete CtP when it was most convenient for them:

I applaud you and do thank you for putting it on the computer instead of doing it all orally. I could do it at my time where I was in a good frame of mind and things were calm here at home. I could do it privately, and I thought it was easy to maneuver. I thought it was really very easily-- yeah, so it was well-done.

Wife, aged 77 years

The CtP tool was easy for SCNs to learn as well:

As far as the mechanics go, the mechanics of Care to Plan I think are easy to learn and navigate. I kind of took a lead role to spend a little bit more time to understand it and then kind of shared it with my colleagues, but I think that the tool itself is built to be easy to learn and to be replicated, so I think that that’s a good feature of the tool.

SCN

### Facilitators to CtP Implementation and Use: Recommendations and Resources Factors

According to the CtP Review Checklist, most caregivers agreed or strongly agreed that CtP provided them with sufficient options for both their needs and that of their people living with dementia; 70% (14/20) of caregivers agreed or strongly agreed that CtP offered them a sufficient number of options to support themselves, and 75% (15/20) of caregivers either agreed or strongly agreed that CtP offered them a sufficient number of options to support their people living with dementia (Table 1). The qualitative interviews complemented these results by adding that caregivers appreciated having a variety of vetted, localized support and resources all in one place. “There’s something for everyone, and not everyone needs everything, but it’s a broad range for everyone” (Wife, aged 74 years). Another noted, “I have not asked for something that they did not have an answer for” (Wife, aged 80 years). The CtP resources empowered caregivers to get help:

For me, getting information really reduced my fear level. It felt like I could guide my family better and then also, to remind me that I needed to take care of myself first before I could take care of my mom and dad. That can’t fade out.

Daughter, aged 52 years

When working with CtP, caregivers were able to explore and connect to supportive resources in their area. One explained how they went from having no resources to having multiple avenues for assistance available in all facets of their caregiving and their sisters’ needs:

I didn’t know nothing about it till I found out about Care to Plan...when I took and called the [local] Area on Agency and everything and they told me if I run into any problems with her and might [be] needing help with a light meal and stuff, and that if she...needed a cell phone, they could get her a cell phone. And the Meals and Wheels was real good, and now I find they could do recreation with her.

Sister, aged 66 years

Overall, caregivers’ reception to CtP implementation was positive: 30% (6/20) of the caregivers noted that the tool could be improved, and 50% (10/20) wished they would have had the opportunity to use the tool sooner in their caregiving role (Table 1).

### Barriers to CtP Implementation and Use: Caregiver Factors

Caregivers generally appreciated the wealth of resources and options suggested by CtP tailoring; however, time constraints made it difficult to take the next steps. Caregivers expressed difficulties in dedicating time to using their resources, feeling overburdened with busy schedules and with their caregiving responsibilities. For instance, one caregiver said:

It’s a matter of sitting down and-- because I’m constantly having to be actively around Dad, and alert of what’s going on. So a lot of times, I sit down and start getting started on something, and then I end up getting sidetracked because I have to get up and intercept him. <laughs> And so...and so a lot of times, I get sidetracked. But yes, I definitely plan on using some of the tools I have learned, most definitely.

Daughter, aged 58 years

With limited time available for themselves, exhausted caregivers do not have the energy for looking through the recommended resources as they would otherwise want to: A wife of one of the people living with dementia explained:

It’s like I’m too tired of thinking to get on the computer and try to research stuff like that. It’s like when I’m not having to do something, I don’t want to do anything else.

Wife, aged 74 years

Caregivers stated that their lack of time to review or access support systems or other resources was exacerbated by the pandemic.

Several caregivers in the early stages of dementia caregiving reported that the resources and recommendations were not currently relevant. However, caregivers recognized their probable utility in the future. One caregiver referred to the resources noting that, “Well, I think that’s going to come in later, the use of these actual features-- the support groups and the respite care” (Wife, aged 74 years).

An unintended consequence of working through the CtP tool with early stage caregivers of people living with dementia was that it provided them with the idea of caregiving needs that may become necessary for their care recipient in the future. On the basis of the suggested resources, one caregiver noted how “...[CtP] just opened my eyes to things that I’ll be facing as time goes on, and how to better understand and cope with it” (Daughter, aged 58 years). Another explained how CtP heightened but also provided solace for their anxiety about their future as a caregiver:

I found it very, very helpful, just the reading itself, the information that was given to me. That’s very helpful. It really is. But as I also said, I guess it kind of frightens me a little bit knowing what might be or probably will be happening in the future...But it’s nice to know that there are people there. Care to Plan is there.

Wife, aged 71 years

Quantitative analysis also identified financial and time constraints as barriers to using services recommended by CtP: 40% (8/20) reported financial constraints, and 45% (9/20) reported time constraints (Table 1). Of the total respondents, 85% (17/20) also reported transportation issues, making it unlikely that they would be able to use the recommendations provided (Table 1). However, 65% (13/20) of the caregivers reported planning to use a service recommended by the CtP (Table 1).

### Barriers to CtP Implementation and Use: SCN Factors

Rapport was also affected by SCN interactions and approach, where some SCNs were naturally engaging and talkative, whereas others were perceived to have approached the use of CtP more rigidly, describing:

I think most of us in this position find it difficult to give a yes/no, black/white [answer]...and I found myself wanting to explain my answers...and the person that did it was very much on--and I understand. I’ve been very much on task and, in a nice way, [the SCN] basically said, “Just answer”...and I had to restrain myself at times to try to explain my answer and not--he wasn’t having any of that, basically, and I understand. I mean, I’ve done research myself, so I understand how that is. It was a little frustrating.

Wife, aged 78 years

According to the CtP Review Checklist, only 45% (9/20) of the respondents reported that their SCN helped them contact a service recommended by CtP (Table 1). Some of these caregivers felt confident enough to walk through the tool themselves but appreciated the guidance of an SCN, just in case they needed them:

Maybe I missed it but I’m a visual person and it would have been really nice to...in hindsight...[hear] “here’s the link why don’t you log on, take a look and then we will set up a call and I’ll go over it with you so that you know what is available here and answer any questions that come up.“

Daughter, aged 48 years

### Barriers to CtP Implementation and Use: CtP System Factors

Caregivers attributed their own technical literacy levels to successfully using the web-based tool on their own but could see how it may be difficult for those not as technically literate. One caregiver said, “I’m very computer-savvy, so I think somebody who isn’t might...have found that difficult” (Wife, aged 78 years). However, for some, internet access was a barrier to completing the tool on their own. As one caregiver noted, “I don’t have Internet service where I live” (Wife, aged 71 years).

### Barriers to CtP Implementation and Use: Recommendations and Resources Factors

Although most caregivers found their CtP recommendations helpful, some were disappointed with their results. According to the CtP Review Checklist, most caregivers were able to find services that met their needs, as well as their relatives, through CtP; 65% (13/20) of the caregivers reported finding a service that met their needs (Table 1). However, 55% (11/20) of the caregivers found a service that met the needs of the people living with dementia (Table 1). Both SCNs and caregivers were frustrated by the lack of a variety of options and tailoring capacity of the CtP tool. Some simply wanted more options to present. For example, 1 caregiver said, “I only got two recommendations and I knew about both of those. I was hoping to get more, more choices of, like, respite care and things that were available” (Wife, aged 75 years). This was reflected in the CtP Review Checklist, in which only 45% (9/20) of the caregivers reported that the resources recommended by CtP were new to them (Table 1). Moreover, 25% (5/20) of the caregivers did not agree that CtP offered them a sufficient number of options to support their people living with dementia (Table 1).

In addition, SCNs and caregivers alike would have preferred further initial questions that helped narrow down the recommendations specific to the current context of care, such as assessing the eligibility of caregivers or people living with dementia for some of the resources beforehand. For example, 1 SCN explained: “I as a navigator don’t ask them on their veteran status and it seems like the VA keeps popping up as a resource and it’s not always appropriate’ (SCN).

Similarly, a caregiver lamented that resources were not available for their particular context saying, “I guess because [name of the person living with dementia] didn’t have issues that they had solutions for, I wasn’t given any solutions for taking care of [name of the person living with dementia]” (Wife, aged 76 years).

Resources were also recommended by caregiver zip codes, which led to some issues in terms of geography and distance to resources. This caregiver explained how the location of the resources recommended was troublesome:

It was more the Southside [city] instead of on this side of the water...[city] is on what’s called the Southside, and it’s across the bay. You have to go across the Chesapeake Bay...and it just didn’t appeal to me.

Wife, aged 77 years

Finally, using CtP during the COVID-19 pandemic shutdowns meant that some resources were unavailable or unusable for caregivers to act on. One caregiver explained “that with the restrictions for group meetings, we couldn’t have any support groups” (Wife, aged 74 years). The pandemic restrictions “kind of put a damper on implementing some of the pieces I wanted to” (Daughter, aged 52 years). Others talked about being more comfortable using the resources once the COVID-19 pandemic is over:

I’ve used some of them, but I’m planning on using more as soon as some of this COVID flack thing. She’s kind of scared to go out right now.

Sister, aged 66 years

## Discussion

### Principal Findings

The objective of this study was to formally explore the feasibility, acceptability, and utility of CtP, an individualized tool for caregivers of people living with dementia that connects them to a diverse array of services that can alleviate caregiver burden and improve other dementia caregiver outcomes. Given that family caregivers receive little support and assistance themselves during their time caregiving [17], CtP is one of the few resources developed for caregivers of people living with dementia that have been implemented in practice [15]. Being one of the few resources for caregivers of people living with dementia, we found that CtP was well-accepted and used by our participants. Caregivers of people living with dementia overwhelmingly agreed that CtP was helpful. The tailored approach used by CtP and the social support provided by SCNs reduced barriers for caregivers of people living with dementia, which is a hallmark characteristic of effective caregiver interventions [9]. Similarly, CtP was further tailored to the needs of caregivers of people living with dementia through linkage with an SCN who provided guidance when using the tool.

Our findings indicated that caregivers of people living with dementia generally appreciated the *single point of entry* for vetted resources and recommendations tailored to them. Quantitative data suggested that the vast majority of caregivers found the tool easy to use. Qualitative data reinforced the helpfulness and convenience of the CtP tool by highlighting its ease of use and its connection to vetted, local resources. Caregivers recognized the value of CtP and wished that they had used it earlier in their caregiving roles.

Alongside the tool, caregivers appreciated having SCNs to discuss barriers to enacting CtP recommendations. Caregivers were able to use their SCNs to navigate community resources, including points of contact, eligibility requirements, and the most effective ways to access services. SCNs also helped caregivers remain accountable for their self-care and well-being. For instance, as SCNs built rapport with caregivers, they were able to make personal connections with caregivers and became their emotional pillars of support, especially during times of overwhelming need. The CtP tool served as a new means by which SCNs can engage family caregivers and help them consider their options both in their current caregiving role and in what that role may require in the future. Thus, CtP serves as another important tool in the *toolbox* of approaches and supports at the disposal of a health care system for families caring for relatives with ADRD.

Although quantitative data suggested that the overwhelming majority of caregivers of people living with dementia found SCNs to be helpful and valued having an SCN available to discuss the recommendations from CtP, qualitative research revealed more nuances in their interactions. Building rapport between SCNs and caregivers of people living with dementia was valuable, as SCNs walked through the CtP tool. The more options CtP presented to caregivers, the more likely they were to feel overwhelmed and in need of SCN support. Less than half of the caregivers reported using their SCN to contact the service recommended by CtP. Having an SCN on standby, available to answer questions, and guide them through the CtP tool (especially during crises or transitions, such as the COVID-19 pandemic) was valuable for caregivers. These results reflect similar studies in which the support of professionals and the simultaneous use of caregiving technologies alleviated challenges during care provision [29,30].

Although quantitative results revealed that most caregivers who used CtP received a sufficient number of options to support themselves or their people living with dementia, caregivers also felt frustrated by the lack of tailored options, as highlighted in the qualitative findings. For example, although caregivers may have received a diverse array of options from the CtP tool, in some cases, they already knew about those options or were unable to accept CtP recommendations because of extraneous constraints such as available time and distance. Some obstacles could be alleviated through more support provided by SCNs and care organizations as well as additional recommended resources on CtP itself. Additional barriers to CtP implementation suggested that the successful use of the CtP tool was dependent on the existing resources of caregivers and highlighted the systematic inequity of today’s digital divide for high-speed internet access [31].

Our study had several limitations. First, the sample was homogenous in terms of race, gender, household income, and marital status; caregivers of people living with dementia were predominantly affluent, White women. With a more educated cohort, our study may have attracted more *tech-savvy* caregivers who are not only more comfortable and receptive to newer technologies but also have more resources to apply such technologies to their lives. Therefore, our results may not be generalizable to a more diverse, less-educated population. As a pilot study, our small sample size inhibited our ability to conduct a more rigorous quantitative analysis of our results. Future research could examine the associations between primary objective stressors, resources, and distress among caregivers of people living with dementia and CtP implementation. Social desirability bias (ie, where a participant may underreport undesirable answers to interview questions) may have also influenced our results. However, our study encouraged participants to report barriers to using the CtP tool. For these reasons, we believe that social desirability bias was curbed. As building rapport between SCNs and caregivers was integral to this pilot study, future research could also explore the personalities of SCNs or their caregivers and how the implementation of CtP (or other innovative technologies) is affected by them.

Within the broader literature of caregiving for people living with dementia, CtP addresses important gaps within the literature. The use of technologies such as CtP offers an innovative, practical, and personalized approach to support caregivers of people living with dementia in health care systems. The application of technology has considerable potential to improve the well-being of caregivers [6,29]. However, SCNs were clearly an integral component of CtP; caregivers appreciated having an expert SCN on standby who understood their own situation. Having a personal connection with caregivers to provide active support over time is another key characteristic of effective interventions to support caregivers of people living with dementia [9]. This study ultimately highlights the importance of complementing both technology and interpersonal connection to support dementia caregivers. Given these conclusions and the 1 month duration, this study encompassed, longer term results that may yield more insight as to how CtP was integrated into routine care and how SCNs provided additional support for caregivers of people living with dementia during their caregiving journey.

The CtP web-based assessment tool with SCNs serving as guides was valuable for caregivers seeking resources to support themselves and their care recipients living with dementia. By sharing the challenges and perspectives of caregivers in their own words, we obtained a richer understanding of their lived experiences. This study highlighted the need for interventions, such as CtP, and the need for financial, time, and transportation constraints to be addressed to improve the utility of caregiver support programs. Although technology-based resources, such as CtP, may overcome certain barriers to care, including knowledge or social support, policy-level changes are necessary to achieve greater equity in caregiving interventions. The lack of high-speed internet, inaccessible transportation, and a strong health care system are all policy-level characteristics unique to a geographic area, and technological resources alone cannot overcome. Further implementation research that is necessary to identify how to best translate and link tailored support assessment tools such as CtP to community programming so that caregivers of people living with dementia are better supported.

## Appendix

Multimedia Appendix 1 Supplementary information about Care to Plan.

## Abbreviations

ADL: activities of daily living
ADRD: Alzheimer disease or related dementia
CES-D: Center for Epidemiological Studies-Depression
CtP: Care to Plan
SCN: senior care navigator
