# Supplementary material for: Delivering Personalized Recommendations to Support Caregivers of People Living With Dementia: Mixed Methods Study
Source: JMIR Aging. 2022 May 3;5(2):e35847. doi: 10.2196/35847 (PMC9067568; doi:10.2196/35847)
Supplement: Multimedia Appendix 1 [file aging_v5i2e35847_app1.docx]

**Multimedia Appendix**

**Table S1. Care to Plan review checklist.**

| **Care to Plan: 21-Review Checklist** | | | | | | |
| --- | --- | --- | --- | --- | --- | --- |
|  | **Strongly Disagree** | **Disagree** | **Neutral** | **Agree** | **Strongly Agree** | **Not Applicable** |
| 1. It was easy to review the Care to Plan tool with Riverside Health | 1 | 2 | 3 | 4 | 5 | 6 |
| 2. The information provided by the  Senior Care Navigator was clear to me | 1 | 2 | 3 | 4 | 5 | 6 |
| 3. The questions I answered during the Care to Plan assessment were easy to understand | 1 | 2 | 3 | 4 | 5 | 6 |
| 4. I was able to understand the service recommendations provided by Care to Plan | 1 | 2 | 3 | 4 | 5 | 6 |
| 5. The person guiding me through Care to Plan was helpful | 1 | 2 | 3 | 4 | 5 | 6 |
| 6. I valued having a Senior Care Navigator available to discuss the recommendations from Care to Plan | 1 | 2 | 3 | 4 | 5 | 6 |
| 7. After using Care to Plan, I was able to find a service that looks as though it will meet my needs | 1 | 2 | 3 | 4 | 5 | 6 |
| 8. After using Care to Plan, I was able to find a service that looks as though it will meet my relative’s needs | 1 | 2 | 3 | 4 | 5 | 6 |
| 9. There are financial constraints to me being able to use the services recommended by Care to Plan | 1 | 2 | 3 | 4 | 5 | 6 |
| 10. There are time constraints to me being able to use the services recommended by Care to Plan | 1 | 2 | 3 | 4 | 5 | 6 |
| 11. I am planning on using a service recommended by Care to Plan | 1 | 2 | 3 | 4 | 5 | 6 |
| 12. The care navigator helped me contact a service recommended by Care to Plan | 1 | 2 | 3 | 4 | 5 | 6 |
| 13. Care to Plan was helpful | 1 | 2 | 3 | 4 | 5 | 6 |
| 14. Care to Plan could be improved | 1 | 2 | 3 | 4 | 5 | 6 |
| 15. I wish I would have completed  Care to Plan sooner | 1 | 2 | 3 | 4 | 5 | 6 |
| 16. Transportation issues make it unlikely that I will be able to use the recommendations provided by Care to Plan | 1 | 2 | 3 | 4 | 5 | 6 |
| 17. Care to Plan provided me with a sufficient number of options to support me | 1 | 2 | 3 | 4 | 5 | 6 |
| 18. Care to Plan provided me with a sufficient number of options to support my relative | 1 | 2 | 3 | 4 | 5 | 6 |
| 19. The resources recommended by Care to Plan were new to me. | 1 | 2 | 3 | 4 | 5 | 6 |
| 20. I would recommend Care to Plan to others in a similar situation | 1 | 2 | 3 | 4 | 5 | 6 |
| 21. I would use Care to Plan again in the future | 1 | 2 | 3 | 4 | 5 | 6 |

Table S2. Facilitators of and barriers to the implementation and use of CtP.

| Facilitator/barrier category | Themes | Description | Supporting quote |
| --- | --- | --- | --- |
| Facilitator | Caregiver factors | CtP appropriately tailored recommendations to caregivers’ needs and context of care | “It broke things down to different types of situations and needs. And in some ways even though I've been living with this, it helped me better understand my situation and my husband's situation...It seemed very thorough, and a logical progression and dealt with not just my husband and his needs, but with me and my needs.” [Wife, aged 76 years] |
| Facilitator | Caregiver factors | CtP was a source of help that caregivers of people living with dementia could turn to with the help of an SCN^a^. | “Help to better define the challenges that caregivers are faced with, that family members are faced with. I think it helps to really provide the proper channeling of resources in the right categories.” [SCN] |
| Facilitator | SCN factors | Caregivers appreciated SCNs when guiding them through the CtP tool. | “Someone who can speak to it personally even though it might not have been exactly the situation in our household, just somebody who totally gets it and how life-changing it is, not just for the person but for the whole family...I really appreciated that part.” [Daughter, aged 58 years] |
| Facilitator | SCN factors | SCNs held caregivers accountable to use CtP. | *“*I actually appreciated the Care Navigator...Just that sometimes when you’re so overwhelmed by everything that’s going on, even though you’re a big person, you still need somebody to kind of take you by the hand and say ‘Let’s get through this. Let’s walk through this and kind of just help you focus’.” [Daughter, aged 56 years] |
| Facilitator | SCN factors | SCNs personalized CtP for caregivers | “It’s that connection of somebody who kind of understands personally what you’re going through, kind of no judgement, someone I felt comfortable enough to share some very candid things with, just the overall frustration and grief and loss you feel. Yeah. So, I thought that was really helpful to have another person who really totally has walked through these steps and several steps ahead. That’s always very helpful.” [Daughter, aged 52 years] |
| Facilitator | SCN factors | SCNs personalized CtP for caregivers | “I think all the content that's in there is quite pertinent, and I think it's information that individuals are really going to need, and I base my answer or comments on the fact that personally I've had to go through this experience of caregiving with my mom, dad and my aunt, and I've actually used the resources, so now as a professional when I'm suggesting the resources and I'm hearing familiar stories of people that are going through struggles with caregiving I can share with them that they're at the right place getting this information.” [SCN] |
| Facilitator | CtP system factors | CtP was user-friendly | “I applaud you and do thank you for putting it on the computer instead of doing it all orally. I could do it at my time where I was in a good frame of mind and things were calm here at home. I could do it privately, and I thought it was easy to maneuver. I thought it was really very easily-- yeah, so it was well-done.” [Wife, aged 77 years] |
| Facilitator | CtP system factors | CtP was user-friendly | “As far as the mechanics go, the mechanics of Care to Plan I think are easy to learn and navigate. I kind of took a lead role to spend a little bit more time to understand it and then kind of shared it with my colleagues, but I think that the tool itself is built to be easy to learn and to be replicated, so I think that that's a good feature of the tool.” [SCN] |
| Facilitator | Recommendations and resources factors | CtP offered enough localized support and resources all in one place for some people | “There's something for everyone, and not everyone needs everything, but it's a broad range for everyone.” [Wife, aged 74 years] |
| Facilitator | Recommendations and resources factors | CtP offered enough localized support and resources all in one place for some people | “For me, getting information really reduced my fear level. It felt like I could guide my family better and then also, to remind me that I needed to take care of myself first before I could take care of my mom and dad. That can’t fade out.” [Daughter, aged 52 years] |
| Facilitator | Recommendations and resources factors | CtP offered enough localized support and resources all in one place for some people | “I didn’t know nothing about it till I found out about Care to Plan...when I took and called the [local] Area on Agency and everything and they told me if I run into any problems with her and might [be] needing help with a light meal and stuff, and that if she...needed a cell phone, they could get her a cell phone. And the Meals and Wheels was real good, and now I find they could do recreation with her.” [Sister, aged 66 years] |
| Barrier | Caregiver factors | Time constraints | “It's a matter of sitting down and-- because I'm constantly having to be actively around Dad, and alert of what's going on. So a lot of times, I sit down and start getting started on something, and then I end up getting sidetracked because I have to get up and intercept him. <laughs> And so... and so a lot of times, I get sidetracked. But yes, I definitely plan on using some of the tools I have learned, most definitely." [Daughter, aged 58 years] |
| Barrier | Caregiver factors | Time constraints | “It's like I'm too tired of thinking to get on the computer and try to research stuff like that. It's like when I'm not having to do something, I don't want to do anything else.” [Wife, aged 74 years] |
| Barrier | Caregiver factors | Irrelevant recommendations or future concerns for caregiving for their people living with dementia | “Well, I think that's going to come in later, the use of these actual features-- the support groups and the respite care.” [Wife, aged 74 years] |
| Barrier | Caregiver factors | Irrelevant recommendations or future concerns for caregiving for their people living with dementia | “I found it very, very helpful, just the reading itself, the information that was given to me. That’s very helpful. It really is. But as I also said, I guess it kind of frightens me a little bit knowing what might be or probably will be happening in the future...But it’s nice to know that there are people there. Care to Plan is there.” [Wife, aged 71 years] |
| Barrier | SCN factors | Differences in SCN interactions affected caregivers’ CtP use | “I think most of us in this position find it difficult to give a yes/no, black/white [answer]...and I found myself wanting to explain my answers...and the person that did it was very much on--and I understand. I’ve been very much on task and, in a nice way, [the SCN] basically said, ‘Just answer’...and I had to restrain myself at times to try to explain my answer and not--he wasn’t having any of that, basically, and I understand. I mean, I’ve done research myself, so I understand how that is. It was a little frustrating.” [Wife, aged 78 years] |
| Barrier | SCN factors | Differences in SCN interactions affected caregivers’ CtP use | “Maybe I missed it but I'm a visual person and it would have been really nice to...in hindsight... [hear] ‘here's the link why don't you log on, take a look and then we will set up a call and I'll go over it with you so that you know what is available here and answer any questions that come up’.” [Daughter, aged 48 years] |
| Barrier | CtP system factors | CtP may be difficult for caregivers who are not technically resourceful | “I’m very computer-savvy, so I think somebody who isn’t might...have found that difficult.” [Wife, aged 78 years] |
| Barrier | CtP system factors | CtP may be difficult for caregivers who are not technically resourceful | “I don’t have Internet service where I live” [Wife, aged 71 years] |
| Barrier | Recommendations and resources factors | CtP did not provide enough recommendations for some caregivers. | “I only got two recommendations and I knew about both of those. I was hoping to get more, more choices of, like, respite care and things that were available.” [Wife, aged 75 years] |
| Barrier | Recommendations and resources factors | CtP did not narrow down recommendations enough; recommendations were inaccessible or did not match their needs | “I as a navigator don’t ask them on their veteran status and it seems like the VA keeps popping up as a resource and it’s not always appropriate.” [SCN] |
| Barrier | Recommendations and resources factors | CtP did not narrow down recommendations enough; recommendations were inaccessible or did not match their needs | “I guess because [name of person living with dementia] didn't have issues that they had solutions for, I wasn't given any solutions for taking care of [name of person living with dementia].” [Wife, aged 76 years] |
| Barrier | Recommendations and resources factors | CtP did not narrow down recommendations enough; recommendations were inaccessible or did not match their needs | “It was more the Southside [city] instead of on this side of the water...[city] is on what's called the Southside, and it's across the bay. You have to go across the Chesapeake Bay...and it just didn't appeal to me.” [Wife, aged 77 years] |
| Barrier | Recommendations and resources factors | CtP did not narrow down recommendations enough; recommendations were inaccessible or did not match their needs | “That with the [pandemic] restrictions for group meetings, we couldn't have any support groups.” [Wife, aged 74 years] |
| Barrier | Recommendations and resources factors | CtP did not narrow down recommendations enough; recommendations were inaccessible or did not match their needs | “[Pandemic restrictions] kind of put a damper on implementing some of the pieces I wanted to.” |
| Barrier | Recommendations and resources factors | CtP did not narrow down recommendations enough; recommendations were inaccessible or did not match their needs | “I’ve used some of them, but I’m planning on using more as soon as some of this COVID flack thing. She’s kind of scared to go out right now.” [Sister, aged 66 years] |

**
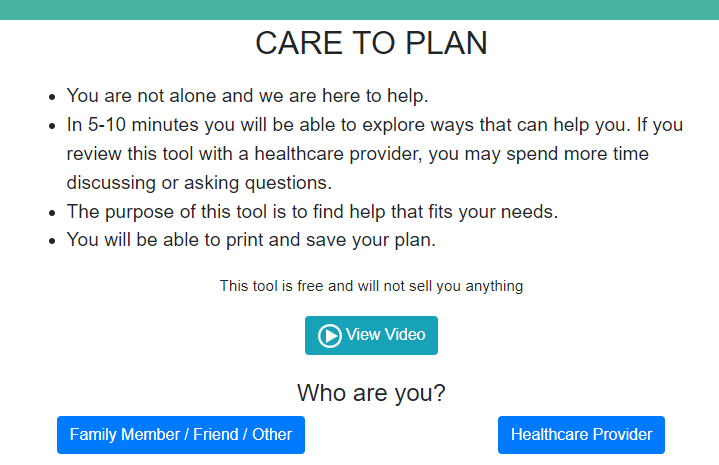
**Figure S1. Care to Plan introductory page


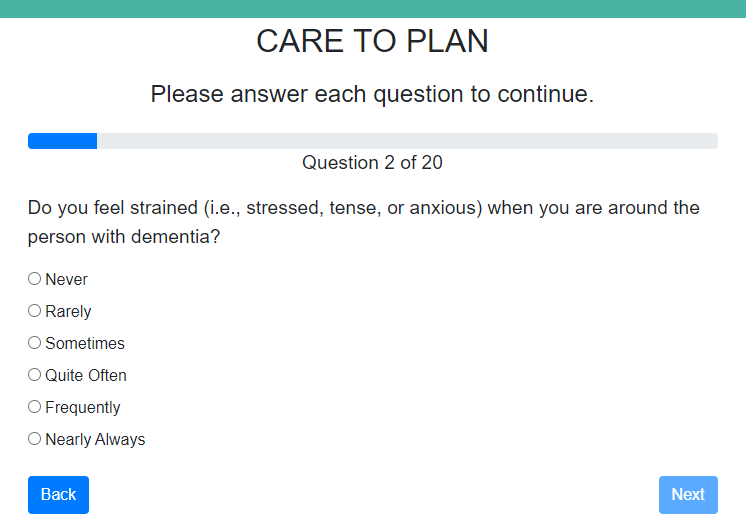

Figure S2. Example question from Care to Plan’s 20-item assessment.


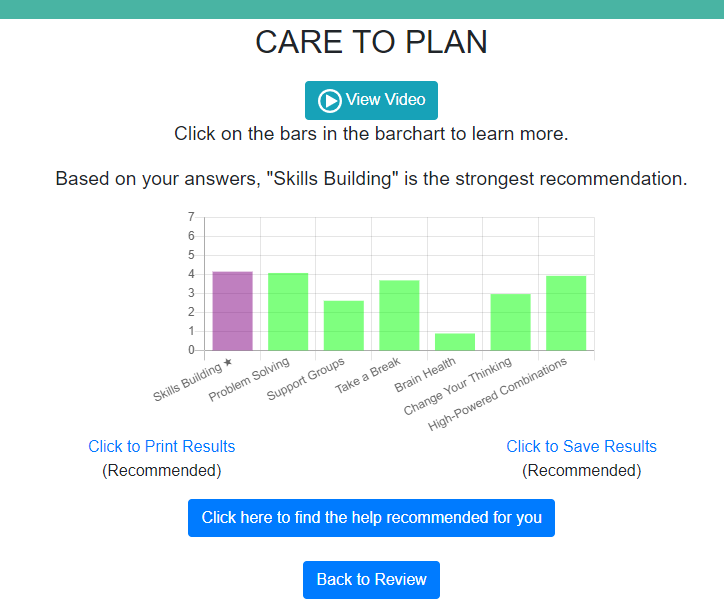


Figure S3. Recommendation types generated by Care to Plan post 20-item assessment completion.
